# Controlling and Optimizing Entropy Production in Transient Heat Transfer in Graded Materials

**DOI:** 10.3390/e21050463

**Published:** 2019-05-03

**Authors:** James Pérez-Barrera, Aldo Figueroa, Federico Vázquez

**Affiliations:** 1 Centro de Investigación en Ciencias (CInC), Universidad Autónoma del Estado de Morelos (UAEM), Av. Universidad 1001, Col. Chamilpa, Cuernavaca 62209, Mexico; 2 CONACyT—Centro de Investigación en Ciencias (CInC), Universidad Autónoma del Estado de Morelos (UAEM), Av. Universidad 1001, Col. Chamilpa, Cuernavaca 62209, Mexico; 3 Energy Engineering Department, Faculty of Mechanical Engineering, Budapest University of Technology and Economics, Müegyetem rkp. 9 D. 208, H-1111 Budapest, Hungary

**Keywords:** functionally graded materials, transient heat transfer, entropy production

## Abstract

This paper presents a numerical analysis of the transient heat transfer problem arising when a functionally graded material is subjected to a fixed temperature difference. Varying the gradation of the system, the thermal performance of the material is assessed both in time-dependent and steady-state conditions by means of temperature profiles and entropy production. One of the main contributions of this paper is the analysis of the system in the transient, from which it is found that the entropy production has a non-monotonic behaviour since maximum and minimum values of this physical quantity could be identified by varying the grading profile of the material. The latter allows to propose an optimization criterion for functionally graded materials which consists of the identification of spatial regions where temperature gradients are large and find thermal conductivity profiles that attenuate those gradients, thus reducing the thermal stresses present inside the material.

## 1. Introduction

Layered materials exhibit discontinuities of thermal and mechanical properties at interfaces due to the bonding of different discrete materials. In particular, at these locations a stress concentration is present which usually leads to delamination, matrix cracking, and adhesive bond separation [1]. The stresses at the interfaces appear from the difference of the thermal expansion coefficients of contiguous layers and the residual stresses due to material processing itself. To solve this problem, materials known as graded materials were conceived [2]. Such materials are formed of two or more constituent phases with a continuously variable composition. It is well known that graded materials possess a number of advantages that make them attractive over conventional layered materials [3]: A potential reduction of in-plane and transverse through-the-thickness stresses, an improved residual stress distribution, enhanced thermal properties, a higher fracture toughness, and reduced stress intensity factors.

Even though one of the main advantages of graded materials is the elimination of stresses at the discontinuites due to the elimination of the interfaces, they do not avoid the appearance of thermal gradients during the transient stage which, in turn, induce thermo-mechanical stresses. Transient and steady state temperature profiles for different graded materials are well studied as evidenced by the many investigations carried out in the last fifteen years [4,5,6,7,8,9,10,11,12,13,14]. Hamza-Cherif et al. [4] analysed the use of the so-called *h-p* version of the finite element method to find the temperature distribution in functionally graded materials. The 2D heat conduction problem was studied and good agreement with analytical solutions was found. The same problem was solved by Sakurai [5] through the use of the moving-particle, semi-implicit method. In this case, the solutions were validated by comparison with finite element solutions, besides analytical solutions, in exponentially and trigonometrically graded systems. A different solution approach based on the Fourier transform was used by Ma and Chen [6] to find theoretical temperature distributions in an exponentially graded material sandwiched between two half planes. A remarkable result indicates how to handle the heat conductivity distribution in the interfaces in order to prevent interfacial fracture problems. Another study by Zhao et al. [7] on 2D heat transport in graded materials was also based on functional transforms (Laplace transform in this case). The authors showed that the method is suitable for finding the temperature distributions of quadratically and trigonometrically, besides exponentially, graded plates. In contradistinction to the previous works, the approach of Rahideh et al. [8] is based on a non-Fourier heat transport equation. Conveniently justified, the model was used to study the effects of the finite speed of wave-like heat transport in multi-layered functional graded materials. Kahn and Aziz [9] characterised the heat transfer from a linear, quadratic, and exponential graded fin through analytical solutions of the heat transport equation of the Fourier type. The shape of the fin cross-section was rectangular, circular, and elliptical. All these cases showed a similar thermal performance. A time non-local description was proposed by Akbarzadeh and Chen in [11] to deal with 1D and 2D heat transport problems in power-law graded systems in different geometries. The formalism was based on the dual phase lag heat conduction, which is another type of the non-Fourier model. It describes in a more suitable way, the causality relations between thermodynamic forces and fluxes. This allows the study of the effects of time delay on the system’s global response. The authors combined Laplace transform and analytical solutions to solve the transport equations. A novel numerical method based on the Lagrange series interpolation was used by Li and Wen [12] to describe temperature distribution in a 2D graded material with heat conductivities depending linearly on spatial variables. The method was properly validated with analytical solutions of the transport equations and its advantages over other methods were discussed. The work by Li et al. [13] focused on solving the transport equations for quadratically and exponentially graded materials through the use of the multiple reciprocity boundary face method. The solutions quickly showed the convergence properties and were validated by comparison with analytical and finite element method solutions. Another research based on the dual phase lag model was performed by Yang et al. [14] on a power-law graded wall. The effects of the magnitude of time delays on the speed of wave-like heat transport through the wall were studied. It was found that big delay times decreases the speed of the thermal wave and that small delay times increases it. This, of course, brings up practical consequences on the construction of furnaces, for instance. Heat waves propagation in graded material also has a great interest in phononics. For instance, it has been shown that rectification of low frequency thermal waves can be achieved in properly graded Si–Ge alloys [15,16,17]. Finally, a recently published review on the development of the research on heat transport problems in functionally graded materials was reported by Swaminathan and Sangeetha [18] with emphasis on numerical solutions methods. In issue 8 of the suggested research, the authors pointed out that:

“Evaluations of most appropriate temperature distribution, for the development of analytical models have to be studied precisely for accurate evaluation of plate deformations.”

From the above discussion, and as far as the present authors know, it can be said that the research on functionally graded materials has mainly focused on the development of reliable numerical solution methods on 2D and 3D systems with linearly, quadratically, trigonometrically, and exponentially grading, and not so on power-law grading, and mostly considered time relaxing processes. The periodically varying operation conditions of many modern technological devices have scarcely been considered. The purposes pursued by the researchers have covered a wide range of applications but have left aside the relationship between irreversibility, inhomogeneity, and performance. This relation is deeply founded on the behaviour of the entropy production in the system which drives the study of the irreversible processes from the point of view of minimum-entropy production principles. As it is known, this condition not only describes fundamental processes in heat transport problems but it serves as an optimisation criterion to reduce thermal stresses, degradation of materials, etc.

Therefore, this work concentrates on the transient analysis of the heat transfer problem in functionally graded materials from the point of view of local and global entropy production in the system. The local analysis will serve as a mean of identifying high thermal gradients inside the material and, consequently, those regions of possible degradation and fracturing of the material. The global analysis, on the other hand, will be used as an optimisation criterion for the gradation of the material in order to ensure an operating regime of minimum entropy production.

The paper starts with a brief introduction about graded materials. Next, the mathematical modelling (governing equations, boundary conditions, grading profile, entropy production, and numerical methodology) is presented. The numerical results and discussion are presented in Section 3 and Section 4, respectively. Finally, the concluding remarks of the paper are presented in Section 5.

## 2. Mathematical Modeling

**Figure 1:**
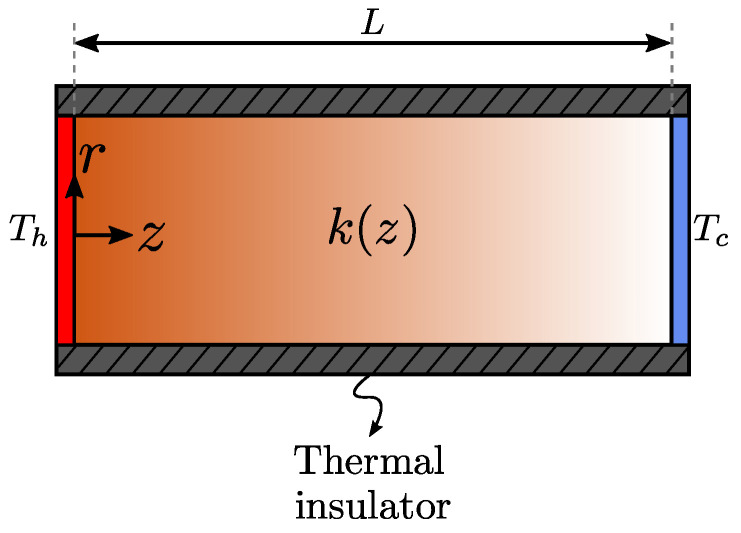
Vertical cross section of a graded cylinder subjected to a temperature difference.

Consider a cylindrical-shaped, thermally-conducting material subjected to a temperature difference $\Delta T=T_{h}-T_{c}$ through its ends, where $T_{h}>T_{c}$. The lateral wall is thermally insulated and the thermal conductivity of the material k(z) varies continuously along the *z* direction between two finite values, $k_{u}$ and $k_{l}$. The total length of the material is *L* and no heat generation occurs inside the material. Figure 1 shows a sketch of the problem at hand.

Application of the energy conservation to the system of interest results in the heat conduction equation [19] which, in dimensionless form reads:

(1) $$\frac{\partial \theta }{\partial t}=\nabla \;\cdot \;\left(k_{z}\left(z\right)\nabla \theta \right),$$

where the axial coordinate *z* and time *t* are normalised by *L* and $\tau =L^{2}/\alpha_{0}$, respectively, and the dimensionless temperature θ is defined as:

(2) $$\theta \left(z,t\right)=\frac{T\left(z,t\right)-T_{c}}{\Delta T}.$$

The thermal diffusivity is denoted by $\alpha_{0}=k_{0}/\rho c_{p}$, where ρ is the mass density, $c_{p}$ is the heat capacity at a constant pressure, and $k_{0}=k_{u}$ is a reference thermal conductivity. Note that, the thermal conductivity $k_{z}\left(z\right)$ depends on the axial position due to the grading of the material.

### 2.1. Initial and Boundary Conditions

It was considered that the graded material is initially at equilibrium with the surroundings and has a uniform distribution of temperature. The assumed initial constant value of temperature is the high temperature in the left side of the graded material, $T_{h}$. The temperature in the right side is suddenly lowered to $T_{c}$ and the transient goes on. The non-dimensional initial condition is written as:

(3) $$\theta \left(z,0\right)=1.$$

The Dirichlet condition:

(4) $$\theta \left(0,t\right)=1,\;\theta \left(1,t\right)=0,$$

constitutes the boundary condition. In practice, the boundary condition is imposed in several ways. For instance, by applying a laser beam to produce a localised heat pulse or by putting the system in contact with solid (liquid) materials on both sides. When this is made through a solid of finite dimensions, which are comparable with those of the system, its temperature must be taken into account and the boundary condition establishes the continuity of the heat flux flowing through the interface, namely,

(5) $$Cd\frac{\partial \theta_{s}\left(0,t\right)}{\partial t}=\kappa \left(0\right)\frac{\partial \theta \left(0,t\right)}{\partial z},$$

where *C*, *d*, and $\theta_{s}$ are the specific heat per unit volume, the thickness, and the temperature of the solid. It is important to mention that in the case described, both temperatures (that of the system and the solid) depend on time. Moreover, the interface acts as a thermal barrier and then there is a discontinuity of temperature given by:

(6) $$\theta_{s}\left(0,t\right)-\theta \left(0,t\right)=-\kappa \left(0\right)R_{K}\frac{\partial \theta \left(0,t\right)}{\partial z},$$

where $R_{K}$ is the Kapitza resistance [20,21,22]. If the graded material is in contact with a liquid, a convection term should be added to the left-hand side in Equation (5). Equations (1), (5), and (6), together with boundary condition Equation (4), are the complete set of equations to solve the heat transport problem. Experiments where the effective heat conductivity of graded materials is measured through time domain thermo-reflectance, have been addressed with Equations (4)–(6) [23,24]. The thermal resistance in solid–solid and solid–water interfaces has also been studied from the microscopic point of view with nonequilibrium molecular dynamics techniques [25,26].

### 2.2. Grading of the Material

Experimental observations [27,28] and other modeling efforts [29,30] have demonstrated that the variation of the properties in graded materials can be approximated as a continuous function varying according to a power law of the *volume fraction*, which, in dimensionless terms is written as:

(7) $$V_{f}\left(z\right)=z^{N},$$

where *N* is the exponent of the power law. The analysed material will be graded for two different cases:

1. Using a low thermal conductivity ($k_{l}$) matrix to be graded with a high thermal conductivity ($k_{u}$) material
  (8) $$k_{z}\left(z\right)=k_{u}\left(1-V_{f}\right)+k_{l}V_{f}.$$
2. Using a high thermal conductivity matrix to be graded with a low thermal conductivity material
  (9) $$k_{z}\left(z\right)=k_{u}V_{f}+k_{l}\left(1-V_{f}\right).$$

**Figure 2:**
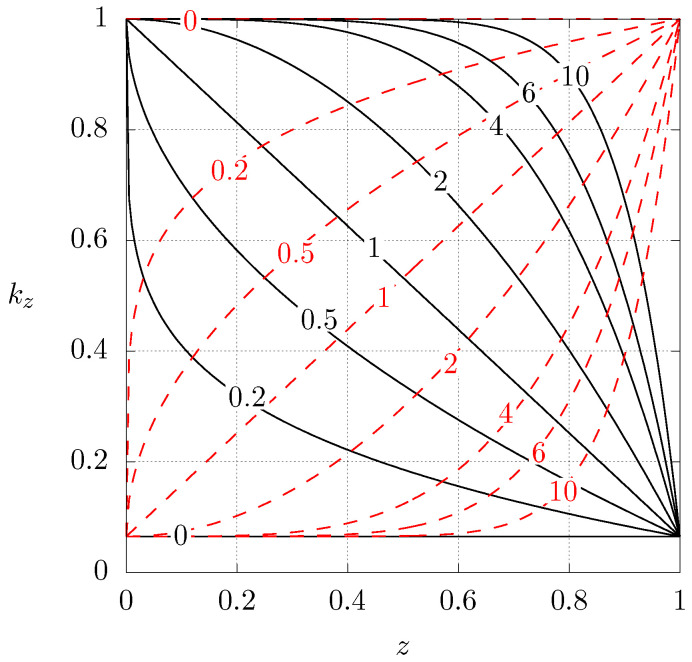
Dimensionless heat conductivity profile as a function of *z* for different values of *N*. Continuous lines, case 1 ($C_{1}$), see Equation (8). Dashed lines, case 2 ($C_{2}$), see Equation (9). In both cases $k_{u}$ is used for normalisation.

Figure 2 shows the dimensionless heat conductivity profiles for the aforementioned case 1 ($C_{1}$) and case 2 ($C_{2}$) as a function of the axial position for different values of *N*. Notice that N=0 corresponds to a homogeneous material with low or high heat conductivity, depending on the case. Starting from N=0, increasing the value of *N* implies that the original material is being gradually replaced by the other one. In the limit N→∞, the original base material has been totally replaced.

### 2.3. Entropy Calculation

Solving Equation (1) yields the temperature as function of position and time, from which different important quantities such as the time elapsed until reaching steady state $t_{S}$, temperature profiles T(z), entropy generation as function of time and space $\dot{s}\left(z,t\right)$, and global entropy production ${\dot{S}}_{g}$ can be obtained. For the entropy generation, the following equation [31] was used:

(10) $$\dot{s}\left(z,t\right)=k\left(z\right)\frac{{\left(\nabla T\right)}^{2}}{T^{2}},$$

which, in dimensionless terms, can be rewritten as:

(11) $$\dot{S}\left(z,t\right)=k_{z}\left(z\right)\frac{{\left(\nabla \theta \right)}^{2}}{{\left[\frac{1}{T_{r}-1}+\theta \right]}^{2}},$$

where $T_{r}=T_{h}/T_{c}$ and $\dot{S}=\dot{s}\;L^{2}/k_{0}$, $k_{0}=k_{u}$ is a reference thermal conductivity. Once the entropy is obtained, it can be integrated in space to yield the average entropy as function of time, and then integrated in time to yield the global entropy production:

(12) $${\dot{S}}_{m}\left(t\right)=\frac{1}{L}\int_{0}^{L}\;\;\;\dot{S}\left(z,t\right)dz,\;\Rightarrow \;{\dot{S}}_{g}=\frac{1}{t_{m}}\int_{0}^{t_{m}}\;\;\;{\dot{S}}_{m}\left(t\right)dt,$$

where $t_{m}$ is the time interval of interest.

### 2.4. Numerical Methodology

In order to capture the physics of the system in the most precise way possible, the equation was solved for the one- and three-dimensional time-dependent cases. The numerical methodology used for the one-dimensional case was the finite volume method as described in [32], whereas for the three-dimensional case, a mixed Fourier Galerkin-Finite Volume method, as proposed by Núñez et al. [33], was used. For both cases, the numerical solutions were found using an in-house FORTRAN numerical code. The govering equation (Equation (1)) is subjected to Dirichlet boundary conditions, the hot and cold ends of the system are denoted by $\theta {|}_{z=0}=1$ and $\theta {|}_{z=1}=0$, respectively. The lateral wall is thermally insulated, that is, $\partial \theta /\partial r{|}_{r=a}=0$, where *a* is the radius of the cylinder. For the three-dimensional case, at the singularity (r=0), the implemented boundary condition consisted of a Neumann-type condition to solve the linear equations and the averaging value of the adjacent cells (sweeping the whole angular direction) was assigned to the temperature $\theta {|}_{r=0}$. For this particular study, the fact that the lateral walls are thermally insulated and that the Dirichlet boundary conditions do not depend on neither the radial nor on the azimuthal coordinate, it is expected that the heat transfer will occur solely in the axial direction, even in the three-dimensional case. The latter was corroborated since there was no appreciable difference between the two models, thus only one-dimensional results are shown hereafter. For all the presented results, the initial condition was taken as a constant, high temperature in the whole system, that is, $\theta {|}_{t=0}=1$. The time integration of the governing heat conduction equation was performed with a second-order Crank–Nicholson time discretisation scheme using a dimensionless time step $\Delta t=1\;\times \;10^{-4}$ and a spatial resolution of $n_{z}=201$ discrete points. The calculation of the global entropy production (Equation (12)) was carried out using the 4-point Newton–Cotes quadrature formula.

## 3. Numerical Results

### 3.1. Validation of the Numerical Code

**Figure 3:**
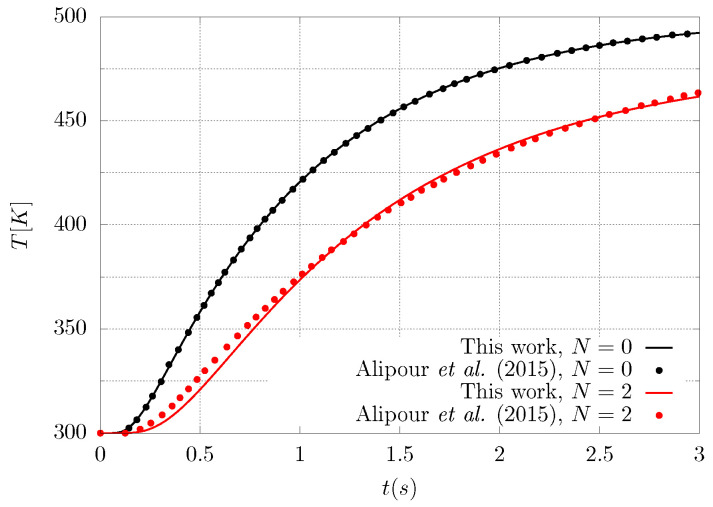
Dimensional temperature at the center of the material as a function of time for two different grading profiles used for validation. Solid lines correspond to the present work whereas the symbols were extracted from Alipour et al. [34].

Alipour et al. [34] studied functionally graded materials in the form of multilayered systems considering space- and temperature-dependent thermomechanical properties of the system and different kinds of boundary conditions, however their research is more focused on the mechanical behaviour of the material (deflection, thermal load, induced axial force, etc.) rather than in the entropy production and its link to material degradation. Despite the study in [34] pointing to a different direction than the present work, some of their results can be used to validate the numerical finite volume code implemented in this paper. Figure 3 shows a comparison between the numerical code used in this work and the generalised differential quadrature methodology followed by Alipour et al. [34], for the temperature at the center of the graded material for two different gradation profiles, namely, N=0 and N=2. It can be seen that there is a good agreement between the two studies for these particular cases and the little differences for N=2 can be explained by the fact that the present work has not considered temperature-dependent properties for the graded material.

### 3.2. Numerical Results for the Present Case

Given the physical setting previously described, imposing the temperature difference through the ends of the material will produce a heat flow from the hot end to the cold one, thus the original distribution of temperature will evolve with time until it reaches steady state conditions. Since the material has a spatial-dependent thermal conductivity and there is no heat generation inside the system, the temperature gradient will depend on the axial coordinate, thus giving rise to different temperature profiles according to the gradation of the material.

**Figure 4:**
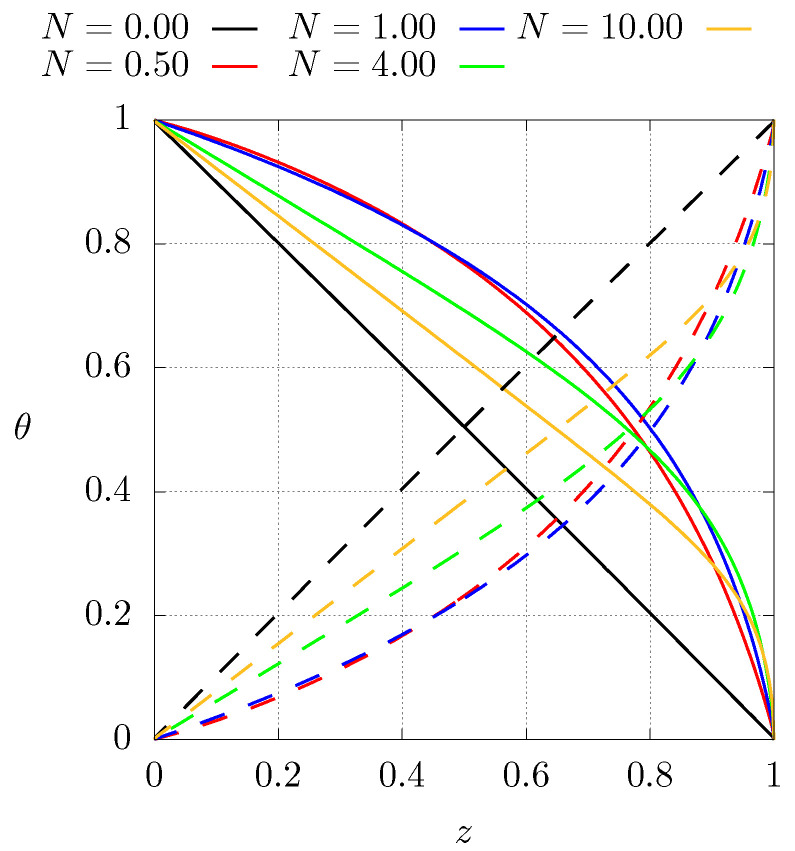
Steady state dimensionless temperature profiles for different values of *N* for case $C_{1}$, see Equation (8). Dashed lines represent the profiles of case 1 with inverted boundary conditions ($C_{1}^{I}$).

Figure 4 shows the steady temperature profiles as function of position for different values of *N* for case 1 ($C_{1}$). The solid lines represent the configuration with boundary conditions described by Equation (4). For the dashed lines, the boundary conditions have been inverted ($C_{1}^{I}$). The case when N=0 corresponds to a homogeneous material with constant thermal conductivity for which the steady temperature profile is a straight line, shows that inverting the thermal conductivity profiles results in lower temperatures than for the homogeneous case. Also note that the higher temperature gradients are located near the cold side of the sample. This implies that the higher mechanical stresses are present near the cold side as well. A further comment related with the entropy production in that region during the transient will be made below.

**Figure 5:**
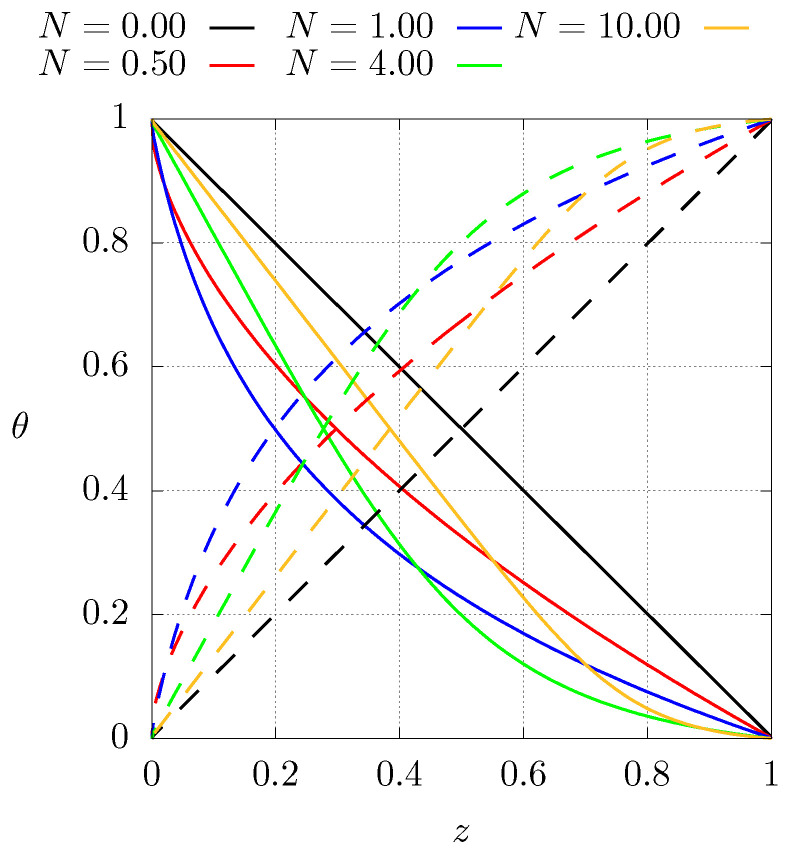
Steady state dimensionless temperature profiles for different values of *N* for case $C_{2}$, see Equation (9). Dashed lines represent the profiles of case 2 with inverted boundary conditions ($C_{2}^{I}$).

Figure 5 shows the same as the previous figure but for case 2. Similarly, the variation of the thermal conductivity profile results in different temperatures than for the homogeneous material, hence, the thermal conductivity profile has a strong influence on the entropy production (Equation (10)). This behaviour can be explained from the fact that, in the absence of heat sources, the heat flux must be constant, but since the thermal conductivity varies as function of position, the temperature gradient must adjust accordingly to meet this condition. Inverting the thermal conductivity profiles results in temperatures that are higher than the homogeneous case.

**Figure 6:**
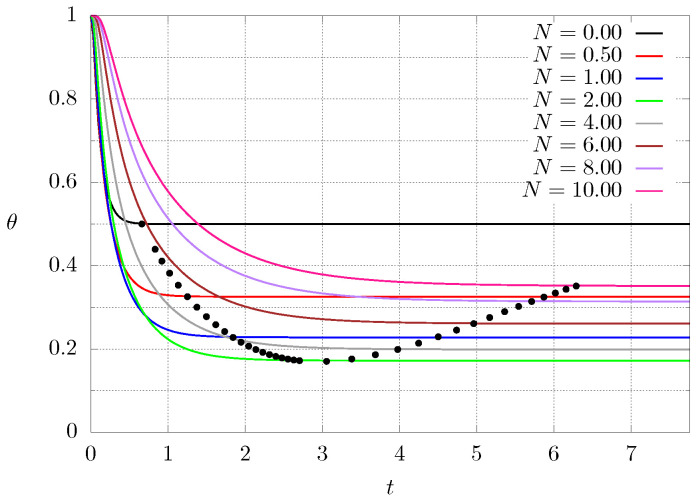
Dimensionless temperature θ at the center of the graded material as a function of dimensionless time for different values of *N* for case $C_{2}$. The black points (•) mark the time needed to reach the steady state $t_{S}$ and the corresponding temperature.

Figure 6 shows the transient behaviour, that is, the temperature at the center of the material as function of time for different values of *N* for case 2. The whole material starts at a high temperature $\theta {|}_{t=0}=1$, then one of the ends is cooled down so that the whole system decreases its temperature. The numerical code starts by calculating the thermal conductivity profile according to Equation (8) or (9), depending on the case. Then Equation (1) is integrated in order to determine the time needed for the system to reach steady state $t_{S}$ (black dots). Since for N=0, the material has a constant thermal conductivity and the temperature profile is a straight line, the steady temperature at the center must be exactly θ=0.5. As N=0 implies high thermal conductivity in homogeneous material, the time needed to reach steady state is small (left-most black point). It can be seen that the grading of the material increases the time interval needed to reach the steady state since the material has lower thermal conductivity is some regions. Along with that, the temperature at the center decreases until reaching a minimum value and then it starts increasing. Since higher values of *N* mean that the original high thermal conductivity material has been replaced by the other lower thermal conductivity one, it is expected that as N→∞, the steady temperature at the center θ→0.5. The behaviour is different when inverting the boundary conditions, for instance, the temperature now has a maximum (instead of a minimum) value and then starts approaching θ→0.5 as N→∞. Similar results can be found for the other case.

**Figure 7:**
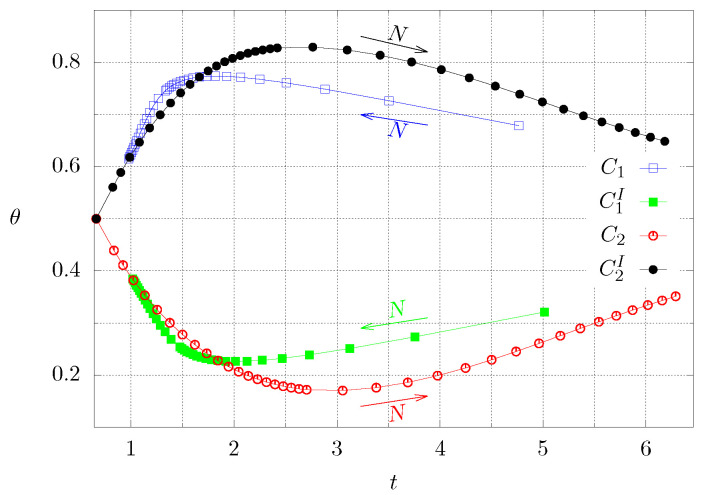
Dimensionless time needed to reach steady state $t_{S}$ and corresponding dimensionless temperature θ at the center for both cases. The superscript *I* means inverted boundary conditions. Each symbol corresponds to a numerical experiment. The continuous lines are a guide to the eye.

Figure 7 shows the time needed to reach steady state for both cases ($C_{1}$ and $C_{2}$) and by inverting the corresponding boundary conditions ($C_{1}^{I}$ and $C_{2}^{I}$). The latter conditions are interesting because many applications in thermal systems, such as materials for construction, are operated under time-periodic boundary conditions. The blue and green lines correspond to case 1, whereas the red and black lines represent case 2. The arrows show the direction of increasing *N*. In both cases it can be observed that the maximum (or minimum) values of the temperature at the center as well as the time interval to reach steady state are not the same if the boundary conditions are inverted.

**Figure 8:**
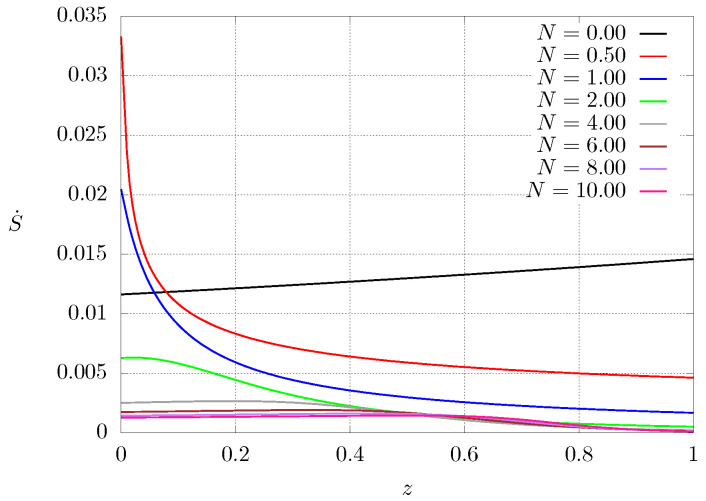
Dimensionless entropy production $\dot{S}$ profiles at steady state as a function of *N* for case $C_{2}$.

Figure 8 shows entropy production as function of the axial coordinate once steady state is reached for case 2. It can be seen that the entropy production is strongly dependent on the spatial position and, thus, on the thermal conductivity profile of the material. If the material is homogeneous (N=0), the thermal conductivity profile is an almost straight line with a small slope, but when the material is graded, the entropy production increases near the heated end where the thermal conductivity is lower, whereas it decreases close to the other end. As a result, regions of high and low entropy production can be identified.

From Figure 5 (solid lines) and Figure 8, it can be seen that the entropy production for steady state conditions is strongly dependent on the temperature profile being higher in regions where the temperature gradient is more pronounced (corresponding to regions where the thermal conductivity is smaller). Similar results can be observed for both configurations by inverting the boundary conditions.

**Figure 9:**
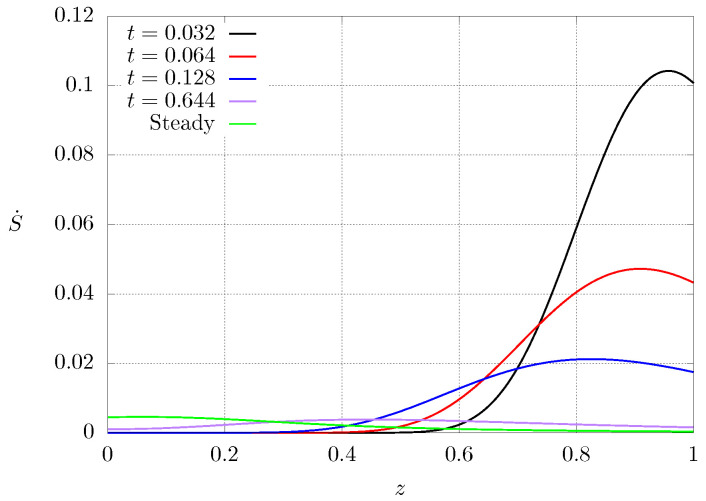
Dimensionless entropy production $\dot{S}$ profiles for different instants of time. Case $C_{2}$ and N=2.5.

However, these entropy production regions are switched during the transient, as seen in Figure 9. This figure shows entropy profiles for different time instants for case $C_{2}$ where N=2.5. It can be observed that at the beginning of the heat transfer process, the entropy profile close to the hot end (low conductivity) is zero because the temperature is kept approximately constant, and the region of highest entropy production lies near the cooled end (high conductivity). As time progresses, the region of high entropy production shifts toward the center (t=0.644) until the highest entropy production lies near the heated extreme (low heat conductivity) when steady state is reached. Since the initial condition is set to a constant, hot temperature, the initial entropy production is zero but, just after imposing the cold temperature at one end of the system, a large temperature gradient arises near the cooled end and this produces high entropy production, as can be observed for t=0.032. It can be seen that the maximum values for the entropy production are larger during the transient, and as time passes, the entropy production becomes smaller very fast due to the fact that the temperature gradient is smoothed out.

**Figure 10:**
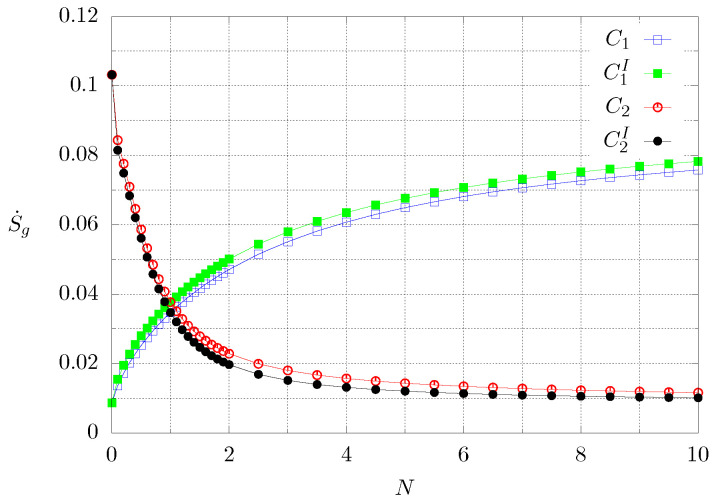
Dimensionless global entropy production for the whole computation time as function of *N*. The superscript *I* means inverted boundary conditions. Each symbol corresponds to a numerical experiment.

Figure 10 shows the global entropy production calculated with Equation (12) for the whole time interval of the computation, for the range 0≤N≤10, for both cases and the inverted boundary conditions. The blue and green lines correspond to the first case and the black and red lines to case 2. One can observe that the larger the value of *N*, the greater the global entropy production for case 1, whereas this behaviour is inverted for case 2. Regardless of the case, the more the material is graded with high thermal conductivity, the greater the entropy production since, for case 1 the thermal conductivity is increased with increasing *N*, whereas for case 2 the heat conductivity is higher for smaller values of *N*. It is also noted that the inversion of the boundary conditions has an effect on ${\dot{S}}_{g}$ in both cases. For the green and red lines, the extreme of the material subjected to the high temperature condition has the lowest heat conductivity, whereas for the blue and black lines, the end with the highest conductivity is held at a high temperature. It can be observed that the configurations for which the global entropy production is greater, corresponds to the heating of the least conductive end of the material.

**Figure 11:**
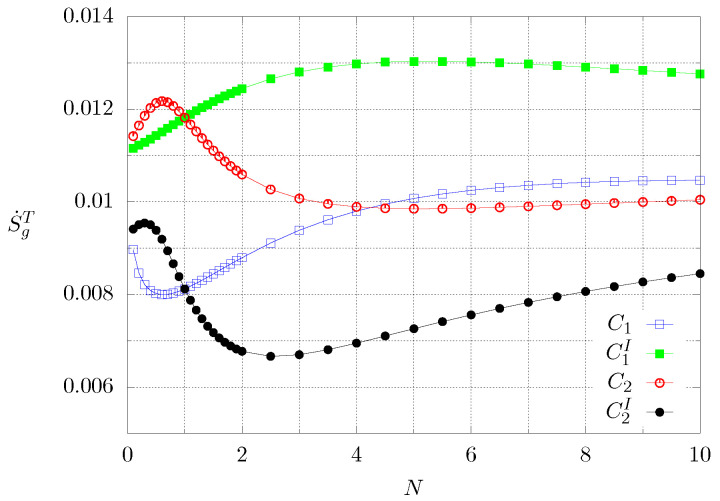
Dimensionless global entropy production until reaching steady conditions as a function of *N*. Each symbol corresponds to a numerical experiment.

Figure 11 shows the behaviour of the global entropy production, but for each value of *N* the time integration was stopped once the steady state was reached. Blue and green lines correspond to case 1, whereas the black and red lines represent case 2. It can be seen that for case 2 there are maximum and minimum values, whereas for case 1 in one configuration there is only a maximum value and for the inverted boundary conditions there is a minimum and possibly a maximum which is not clearly seen because of the plotting resolution. Comparing cases $C_{1}$ and $C_{2}$, the first case shows a lower global entropy production ${\dot{S}}_{g}$ for N<4, and higher ${\dot{S}}_{g}$ for N>4. At N≈4.2, both cases show the same value for ${\dot{S}}_{g}$. A similar behaviour can be seen if cases $C_{1}$ and $C_{2}$, with their corresponding inverted boundary conditions, are compared.

For transient conditions, the entropy production is different if the calculation is stopped once steady state is reached as can be noted by comparing Figure 10 and Figure 11. The first one shows the global entropy production for a fixed time interval which is the same for all cases, whereas for the other figure, the entropy was calculated only for the time interval the transient behaviour lasts, which differs for every conductivity gradient. On the one hand, it can be noted that the global entropy has a monotonous behaviour as the grading of the material increases, but for the transient, the behaviour is non-monotonous since there are maximum and minimum values of entropy production. This points out to the idea that there are optimal heat conductivity profiles for materials that are subjected to time-dependent boundary conditions, like construction materials used for buildings and housing or thermoelectric materials.

## 4. Discussion

The one- and thee-dimensional models agree qualitatively and quantitatively due to the symmetry of the problem, which somehow validates the numerical results, and thus the problem can be analysed as one-dimensional. Moreover, our numerical code was tested satisfactorily with numerical transient results from the heat transfer problem in a multilayered system previously analysed [34], which validates our transient analysis.

In our problem case, it was found that the duration of the transient is dependent on the grading profile so that the more the material is graded with low heat conductivity, the more time was needed to reach steady state. It was also found that the temperature gradient was strongly dependent on the spatial coordinate and this has a repercussion on the entropy production, for instance, regions of high and low entropy production arise and thus, the region where entropy production was larger corresponded to low heat conductivity of the material. Surprisingly, the low and high entropy production regions were switched during the transient. It was demonstrated that the analysis of the transient heat transfer was very important since the behaviour of the entropy production was non-monotonous, having maximum and minimum values of these physical quantity depending on the grading profile. On the contrary, when the time interval was the same for all the different grading profiles, then entropy production had a monotonic behaviour but was affected if the boundary conditions were inverted.

It was interesting to calculate the ratio *R* of the global entropy produced till the steady state was reached (${\dot{S}}_{g}^{T}$) to the global entropy produced in whole computational time (${\dot{S}}_{g}$). Table 1 shows the result for the cases where there exist a minimum or a maximum for certain values of *N* accordingly with Figure 11. The second column of the table indicates if the extreme is a minimum or a maximum. The corresponding value of *N* can be seen in the third column. The fourth column shows the value of the global entropy produced till the steady state is reached. The following column contains the corresponding global entropy produced during the whole computational time and, finally, the last column shows the ratio of these two values.

Several comments can follow from Table 1. Starting with the assumption that the system operates in a stationary state, the entropy produced in the whole process is a minimum. Therefore it should be desirable to use those materials with big values of ratio *R*. As seen from the fifth column of Table 1, the materials showing a minimum in the total global entropy production are $C_{1}$ (N=0.6), $C_{2}$ (N=5), and $C_{2}^{I}$ (N=2.5). From them, material $C_{2}$ (N=5) should be selected (R=0.7). On the other hand, if the system operates in the transient, the material $C_{1}$ (N=0.6) should be used (R=0.32). It is worth comparing the functioning of this last material with material $C_{2}^{I}$ (N=0.3) which has a total global entropy production of 0.062 (dimensionless units) from which 0.0093 is produced during the transient state and 0.0527 in the stationary state. On the other hand, material $C_{1}$ (N=0.6) produces a total of 0.025 from which 0.008 are produced during the transient and 0.017 during the stationary state. Operating in the transient state, both of materials had a similar thermal performance. In contradistinction, when in the stationary state, material $C_{1}$ (N=0.6) is preferable to material $C_{2}^{I}$ (N=0.3) since the former produced about 32% of that of the second one.s

## 5. Concluding Remarks

A numerical study of the thermal behaviour of functionally graded materials using different thermal conductivity profiles and inverting the imposed boundary conditions was performed. Even though there exist studies about the improvement of thermal efficiency using functionally graded materials in the past fifteen years, e.g., [35], an analysis relating it with entropy production in transient state has not been published, to the best of our knowledge. Several physical aspects were discussed, namely, (i) time needed to reach the steady state, (ii) temporary entropy production profiles, (iii) total entropy produced in the transient, and (iv) global entropy production.

Since entropy production can be linked with the degradation of the material, the main conclusions refer to the relationship of volume fraction-entropy production that allowed to identify volume fractions distributions involving smaller global entropy and found the optimal grading profile for particular applications where the heat transport was time dependent i.e., materials for buildings and housing or thermoelectrics where the thermal systems are operated with time-dependent (periodic) boundary conditions.

## Figures and Tables

**Table 1:** Numerical results for maximum and minimum values of entropy production during the transient and for the whole time interval.

Material	Min/Max	*N*	${\dot{S}}_{g}$	${\dot{S}}_{g}^{T}$	$R={\dot{S}}_{g}/{\dot{S}}_{g}^{T}$
$C_{1}$	Min	0.6	0.008	0.025	0.32
$C_{1}^{I}$	Max	5.5	0.013	0.069	0.19
$C_{2}$	Max	0.6	0.0122	0.053	0.23
$C_{2}$	Min	5	0.0098	0.014	0.7
$C_{2}^{I}$	Max	0.3	0.0095	0.062	0.15
$C_{2}^{I}$	Min	2.5	0.0067	0.017	0.39
